# Forecasting the Effects of the New SARS-CoV-2 Variant in Europe

**DOI:** 10.1155/2021/5553240

**Published:** 2021-05-04

**Authors:** Iván Area, Juan J. Nieto

**Affiliations:** ^1^Universidade de Vigo, Departamento de Matemática Aplicada II, E.E. Aeronáutica e do Espazo, Campus As Lagoas-Ourense, Ourense 32004, Spain; ^2^Instituto de Matemáticas, Universidade de Santiago de Compostela, Santiago de Compostela 15782, Spain

## Abstract

Due to the emergence of a new SARS-CoV-2 variant, we use a previous model to simulate the behaviour of this new SARS-CoV-2 variant. The analysis and simulations are performed for Europe, in order to provide a global analysis of the pandemic. In this context, numerical results are obtained in the first 100 days of the pandemic assuming an infectivity of 70%, 56%, and 35%, respectively, higher for the new SAR-CoV-2 variant, as compared with the real data.

## 1. Introduction

The pandemic of COVID-19 has caused different crises at many levels, health, economy, financial, and employment, just to cite some of them.

Since the declaration of COVID-19 as a global pandemic by the World Health Organization, various territories have been taking various measures that, to a greater or lesser extent, have helped reduce the number of new daily cases of infected people. In this work, we shall refer to this as basic quantity since from that number, it is possible to deduce, for example, hospital pressure based on new daily cases accumulated at 15 days, or the number of ICU beds required [1]. This has been one of the cornerstones in the decision making on COVID-19. As soon as the saturation problems began in the hospitals, the measures became, and will become, more drastic. Reciprocally, as health parameters improved, societal measures were withdrawn. But, these measures were being adopted either by the territories or by the member states of the European Union, far from a global vision of the problem.

During these months, many authors have focused their analysis on specific territories, providing appropriate curves [2]. In the specific case of Galicia (northwest Spain), the curve dropped to zero over a period in the late July 2020. This is the case of many other European territories. Nevertheless, mobility between zones and the fact that different confinements were decreed at different times led to certain euphoria in some regions, far from the reality globally.

A new SARS-CoV-2 variant that contains a series of mutations has been described in the United Kingdom (UK) and become highly prevalent in London and southeast England [3], and this variant is 56% more transmissible than other strains, according to the study by the Centre for Mathematical Modelling of Infectious Diseases at the London School of Hygiene and Tropical Medicine [4]. A new SARS-CoV-2 variant is spreading fast in the UK with over 1,400 cases since September. This new version of SARS-CoV-2—named the B.1.1.7 lineage—is spreading in the U.K. and possibly beyond [5]. Due to the emergency of this new situation, we have reported to the European Commission to notice the different behaviour and infectivity of the new strain at the time of writing and submitting this manuscript (December 27, 2020).

Some time ago, we warned of the need to take global action instead of local as the problem we have reached that dimension, as stated by the World Health Organization.

**Figure 1:**
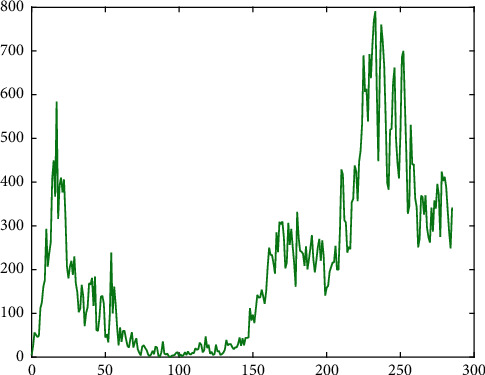
Number of new daily infected individuals in Galicia, starting March 13, 2020.

If we summarize the first wave, we can see that, from the beginning to the 100th day, we have a fairly rapid rise and then a longer decline in time (see Figure 1 with data from Galicia, an autonomous community of Spain which is geographically located in the northwest Iberian Peninsula, having a population of about 2,700,000 and a total area of 29,574 km^2^). The second wave began to form between days 150 and 200 with a very rapid rise and again the descent (see, again, Figure 1).

**Figure 2:**
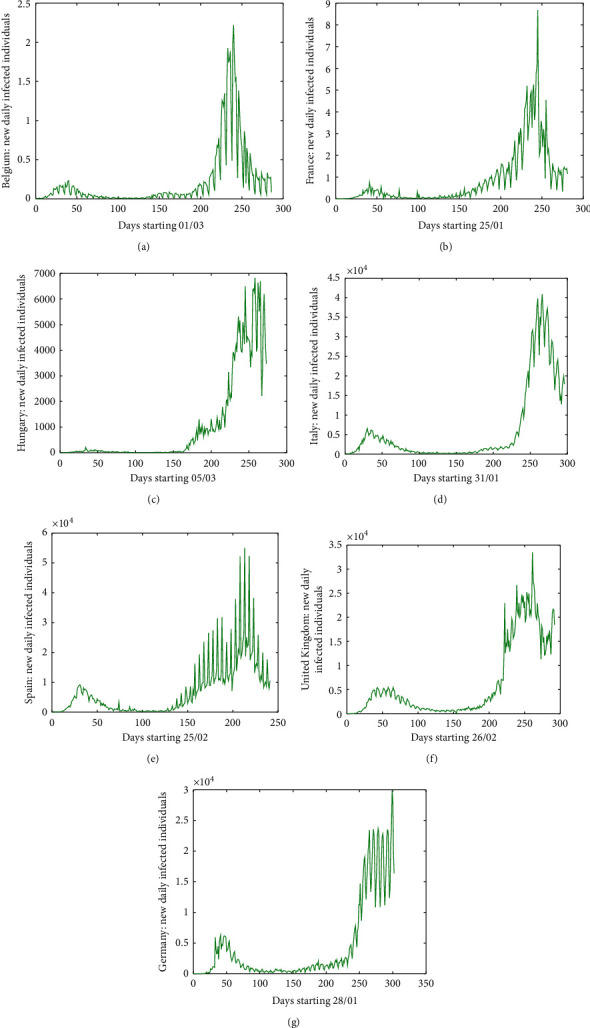
Number of new daily infected individuals in (a) Belgium, (b) France, (c) Hungary, (d) Italy, (e) Spain, (f) the United Kingdom, and (g) Germany.

Similar figures could be added for any other territory, region, or country, and the number of picks is remarkable due to many problems and methodologies in collecting the data. In this direction, we have chosen some of them: Belgium, France, Hungary, Italy, Spain, and the United Kingdom (see Figure 2), but many others could be added in the same direction. For instance, in some countries, real data are not being published during weekends. Moreover, during the worst days of the pandemic, under some circumstances, it has been given priority to handle ill people in front of counting the new infected individuals. In order to minimize these picks, it might be much better to have, e.g., a mean of 5 or 7 days, which give much more smooth curves and realistic data.

**Figure 3:**
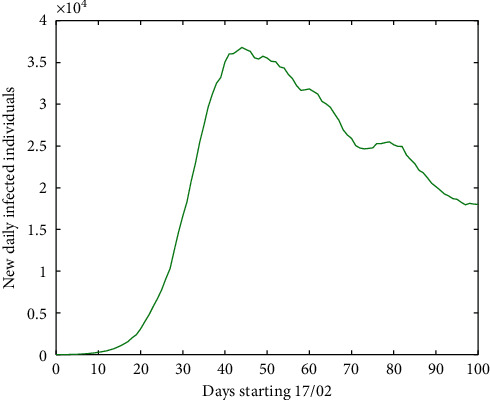
Number of new daily infected individuals in Europe, starting 17 February 2020 and computing the first 100 days with the mean of the seven previous days.

As mentioned before, in many territories, zero new daily infected individuals was attained. On the other hand, if we look at the graph at the European level, it is observed that we never reached zero in new infected people. Rather, we have come a long way from that amount, as can be seen in Figure 3, where we show the first 100 days starting in March and in which for each day, we have computed the mean of the previous 7 days.

**Figure 4:**
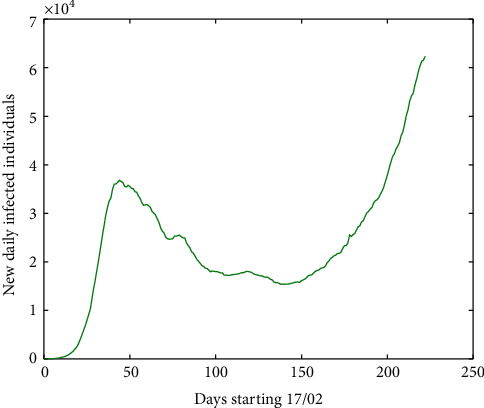
Number of new daily infected individuals in Europe, starting 17 February 2020 up to 30 September 2020.

From Figure 3 (February–May 2020), we can observe the rapid rise and the long time required for the number of new cases to be gradually reduced. If we increase the time horizon until September, we obtain Figure 4 where it is clearer that the absolute zero was not attained.

It is important to notice here that, during the pandemic, different policies have been adapted at different territories, and moreover, the influence of an individual's behavioural response due to the information regarding proper precautions is extremely important. In this direction, in [6], a compartmental mathematical model of the SEIRS type to analyze the spread of the COVID-19 pandemic was introduced. Moreover, it is also important to emphasize that the numbers of the first wave appear to be lower than those of the second which, however, is not true as in the first wave, only symptomatic people were tested (where it was possible).

## 2. Numerical Simulations for the New Strain

Theoretically, a newer strain may emerge in the population when a preexisting strain has reached equilibrium [7].

The need of integrated science to fight the COVID-19 pandemic is apparent [8]. Different approaches are necessary ranging from mouse models [9] or behavioural response of the population [10] to mathematical and simulation models [11].

The results in [12] reveal the intrahost genomic diversity and plasticity of SARS-CoV-2, pointing out genomic regions that are prone to alterations. This may be related to the recent emergence of a new SARS-CoV-2 variant, mainly in the UK.

The emergence of a new SARS-CoV-2 variant leads to thinking about what the situation would have been and will be with a higher level of infection. There are not yet enough studies on this mutation of the virus. Therefore, any contribution may help. It is also true that the measures of social distancing in the United Kingdom are not comparable to those we have in other European zones and Galicia. But, it is true that if it is confirmed that this new strain is much more contagious and also affects adults and infants or children, we would have absolutely terrifying curves of new infected cases. This is the main aim of this work.

We use some corroborated models to compare the infectivity of the original COVID-19 and other strains with more infectivity.

**Figure 5:**
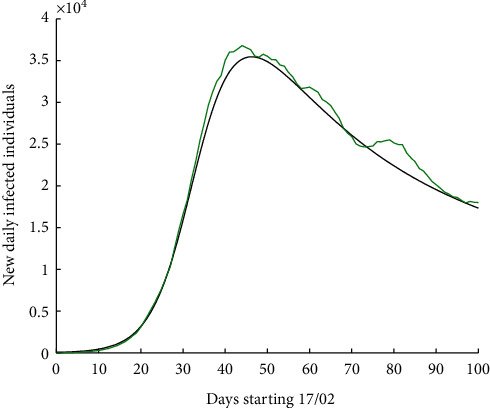
Number of new daily infected individuals in Europe, starting 17 February 2020 for 100 days. Real data are shown in green colour, while the result of the numerical simulations using fractional differential equations is shown in black colour.

According to our mathematical compartmental models, we were able to predict how the first wave of the pandemic was in China and Galicia [1, 2]. Moreover, by using a system of fractional differential equations based on the same model, we have been able to predict that of Portugal, Galicia, and Spain [13–15]. By applying the latter model to the European context, we obtain Figure 5 for Europe.

**Figure 6:**
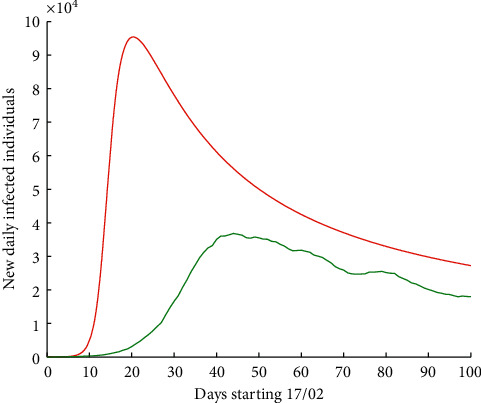
Number of new daily infected individuals in Europe, starting 17 February 2020 for 100 days. Real data are shown in green colour, while the result of the numerical simulations of the hypothetic curve with 70% more of infectivity is shown in red colour.

If we now apply a correction factor by increasing infectivity, say by 70%, we would have Figure 6, where in red is the new wave for Europe. It would undoubtedly imply the saturation of health systems in different countries. In this graph, the maximum would be attained in 21 days with 95,317 new infected individuals. It is also important to notice the highly increasing slope of the curve that would generate a number of people at hospitals and ICUs.

**Figure 7:**
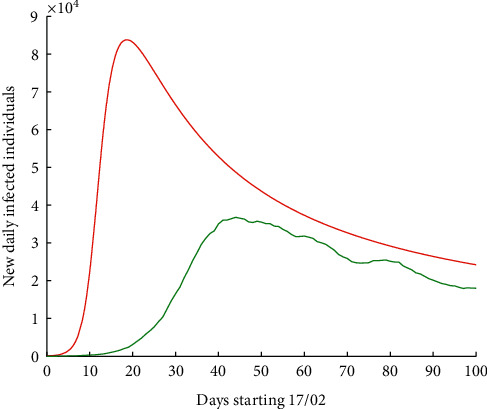
Number of new daily infected individuals in Europe, starting 17 February 2020, for 100 days. Real data are shown in green colour, while the result of the numerical simulations of the hypothetic curve with 56% more of infectivity is shown in red colour.

If we apply a correction factor by increasing infectivity by 56% [4], we obtain Figure 7, where in red would be the new wave for Europe, which undoubtedly would involve again the saturation of health systems in different countries, since the maximum would be attained in 21 days with 83,172 new infected individuals.

**Figure 8:**
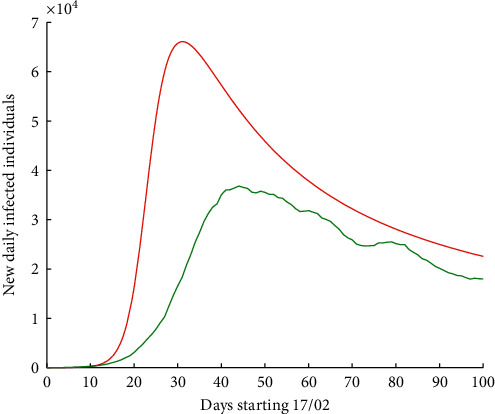
Number of new daily infected individuals in Europe, starting 17 February 2020 for 100 days. Real data are shown in green colour, while the result of the numerical simulations of the hypothetic curve with 35% more of infectivity is shown in red colour.

Finally, we now apply a correction factor by increasing infectivity by 35%. We have Figure 8, where, again, in red colour is the new wave for Europe, with the saturation of health systems in different countries with a maximum attained of 65,875 in 32 days.

## 3. Conclusions

In order to be able to take adequate measures, we simulate the new highly infective strain of SARS-CoV-2 at a European level.

Due to the emergence of a novel SARS-CoV-2 variant, VOC 202012/01, and in order to be able to take adequate measures, we simulate the new highly infective strain of SARS-CoV-2 at the European level.

Assuming the infectivity increment of 70%, the situation will be dramatic. With an increment of only 35%, the consequences are also severe. With a 56% increment, the situation may be also critical with a maximum of new infected individuals in about a month.

Optimal control with vaccination constraints have been analyzed for several diseases such as Zika [16], dengue [17], or Ebola [18]. One of the major problems is to decide the different classes of population to be vaccinated, taking into account their vulnerability as well as the number of available vaccines. Three main problems can be taken into consideration: the number of required beds at intensive care units, the number of hospitalized individuals, and of course, the number of dead individuals. One possible option is to conduct vaccination by age groups, starting from older people. After this submission has been done, the vaccination program started in Galicia, a region in the northwest of Spain. More precisely, before the vaccination, in Galicia, with a total population of 2,700,000 people, 5.5% of them more than or equal to 80 years of age, they represented 8% of new individuals at ICUs, 38% of new hospitalized individuals, and 9% as newly infected individuals on average in December 2020. By 28 December 2020, the vaccination program started, and up to now, it has reached about 4.5% of the total population, mainly to the oldest individuals, which has not yet finished. During the vaccination program (not yet finished), these values have reduced up to 4% (ICUS), 27% (hospitalization), and 7% (newly infected individuals). Also, we have reduced globally from 14 new individuals at ICUs (on average in January 2021) to less than 7 (on average in March 2021).

Of course, the influence of vaccination will be taken into account in a future work and implemented into the model. Without doubt, appropriate measures and social policies should be implemented.

## Data Availability

Data are available on request to the corresponding author.

## Additional Points

*Dissemination to Participants and Related Patient and Public Communities*. There are no plans to disseminate the results of the research to study participants. Study results will be shared with the public via press release, social media, and conference presentations. Patients who had previously had COVID-19 and their families were not involved in setting the research or the outcome measures.
